# Assessing the WHO-UNICEF primary health-care measurement framework; Bangladesh, India, Nepal, Pakistan and Sri Lanka 

**DOI:** 10.2471/BLT.23.290655

**Published:** 2024-04-30

**Authors:** Neha Purohit, Navneet Kaur, Syed RM Zaidi, Lalini Rajapaksa, Malabika Sarker, Shiva R Adhikari, Shankar Prinja

**Affiliations:** a Department of Community Medicine and School of Public Health, Post Graduate Institute of Medical Education and Research, Sector-12, Chandigarh 160012, India.; b Ministry of National Health Services, Regulations and Coordination, Islamabad, Pakistan.; c Faculty of Medicine, University of Colombo, Colombo, Sri Lanka.; d BRAC P James Grant School of Public Health, BRAC University, Dhaka, Bangladesh.; e Central Department of Economics, Tribhuvan University, Kirtipur, Nepal.

## Abstract

**Objective:**

To assess the availability of information on indicators of the World Health Organization and United Nations Children’s Fund primary health-care measurement framework in Bangladesh, India, Nepal, Pakistan and Sri Lanka and to outline the opportunities for and challenges to using the framework in these countries.

**Methods:**

We reviewed global and national data repositories for quantitative indicators of the framework and conducted a desk review of country documents for qualitative indicators in February–April 2023. We assessed data sources and cross-sectional survey tools to suggest possible sources of information on framework indicators that were not currently reported in the countries. We also identified specific indicators outside the framework on which information is collected in the countries and which could be used to measure primary health-care performance.

**Findings:**

Data on 54% (32/59) of the quantitative indicators were partially or completely available for the countries, ranging from 41% (24/59) in Pakistan to 64% (38/59) in Nepal. Information on 41% (66/163) of the qualitative subindicators could be acquired through desk reviews of country-specific documents. Information on input indicators was more readily available than on process and output indicators. The feasibility of acquiring information on the unreported indicators was moderate to high through adaptation of data collection instruments.

**Conclusion:**

The primary health-care measurement framework provides a platform to readily assess and track the performance of primary health care. Countries should improve the completeness, quality and use of existing data for strengthening of primary health care.

## Résumé

**Objectif:**

Étudier la disponibilité des informations concernant les indicateurs du cadre d'évaluation des soins de santé primaires de l'Organisation mondiale de la Santé et du Fonds des Nations Unies pour l'enfance au Bangladesh, en Inde, au Népal, au Pakistan et au Sri Lanka, mais aussi présenter les opportunités et enjeux de l'application d'un tel cadre dans ces pays.

**Méthodes:**

Nous avons exploré les référentiels de données nationaux et internationaux à la recherche d'indicateurs liés au cadre, puis nous avons effectué un examen préliminaire des documents de chaque pays pour y trouver des indicateurs qualitatifs entre février et avril 2023. Nous avons évalué la provenance des données et les outils d'enquête transversale en vue de suggérer des sources potentielles d'informations sur certains indicateurs relatifs au cadre qui n'apparaissaient pas encore dans les pays susmentionnés. Nous avons par ailleurs identifié des indicateurs spécifiques en dehors du cadre, sur lesquels les pays collectent des informations et qui pourraient servir à mesurer les performances des soins de santé primaires.

**Résultats:**

Des données sur 54% (32/59) des indicateurs quantitatifs étaient partiellement ou entièrement disponibles dans les différents pays, allant de 41% (24/59) au Pakistan à 64% (38/59) au Népal. Des examens préliminaires de documents propres à chaque pays ont permis de recueillir des informations sur 41% (66/163) des sous-indicateurs qualitatifs. Il était plus facile d'obtenir des précisions sur les indicateurs de moyens que sur les indicateurs de processus et de résultats. Enfin, le degré de faisabilité de l'acquisition d'informations sur les indicateurs non mentionnés était modéré à élevé, à condition d'adapter les instruments de collecte de données.

**Conclusion:**

Le cadre d'évaluation des soins de santé primaires offre une plateforme permettant d'évaluer et de suivre aisément les performances en la matière. Les pays devraient améliorer l'exhaustivité, la qualité et l'utilisation des données existantes pour renforcer les soins de santé primaires.

## Resumen

**Objetivo:**

Evaluar la disponibilidad de información sobre los indicadores del marco de medición de la atención primaria de salud de la Organización Mundial de la Salud y el Fondo de las Naciones Unidas para la Infancia en Bangladesh, India, Nepal, Pakistán y Sri Lanka, y exponer las oportunidades y los desafíos que plantea la aplicación del marco en estos países.

**Métodos:**

Se revisaron los repositorios de datos mundiales y nacionales para los indicadores cuantitativos del marco y se realizó una revisión preliminar de los documentos de los países para los indicadores cualitativos entre febrero y abril de 2023. Se evaluaron las fuentes de datos y las herramientas de encuestas transversales para sugerir posibles fuentes de información sobre los indicadores del marco que actualmente no se comunican en los países. También se identificaron indicadores específicos ajenos al marco sobre los que se recopila información en los países y que se podrían aplicar para medir los resultados de la atención primaria.

**Resultados:**

Los datos sobre el 54% (32/59) de los indicadores cuantitativos estaban parcial o totalmente disponibles para los países, oscilando entre el 41% (24/59) de Pakistán y el 64% (38/59) de Nepal. La información sobre el 41% (66/163) de los subindicadores cualitativos se pudo obtener mediante revisiones preliminares de documentos específicos de cada país. La información sobre los indicadores de insumos fue más fácil de obtener que sobre los indicadores de procesos y productos. La viabilidad de adquirir información sobre los indicadores no declarados fue de moderada a alta mediante la adaptación de los instrumentos de recopilación de datos.

**Conclusión:**

El marco de medición de la atención primaria proporciona una plataforma para evaluar y seguir fácilmente el rendimiento de la atención primaria. Los países deben mejorar la exhaustividad, la calidad y el uso de los datos existentes para reforzar la atención primaria.

## ملخص

**الغرض:**

تقييم مدى توفر المعلومات عن مؤشرات إطار العمل الخاص بقياس الرعاية الصحية الأولية لمنظمة الصحة العالمية، وصندوق منظمة الأمم المتحدة للطفولة، في كل من بنغلاديش والهند ونيبال وباكستان وسري لانكا، وتحديد الفرص والتحديات التي تواجه استخدام إطار العمل هذا في تلك الدول.

**الطريقة:**

قمنا بمراجعة مستودعات البيانات العالمية والوطنية لفحص المؤشرات الكمية لإطار العمل، كما قمنا بمراجعة مكتبية للوثائق القطرية للمؤشرات النوعية خلال الفترة من فبراير/شباط إلى أبريل/نيسان 2023. وقمنا بتقييم مصادر البيانات وأدوات المسح متعددة القطاعات بهدف اقتراح مصادر ممكنة للمعلومات عن مؤشرات إطار العمل هذا، والتي لم يتم الإبلاغ عنها حاليًا في الدول. قمنا أيضًا بتحديد مؤشرات محددة خارج إطار العمل، والتي يتم وفقًا لها جمع المعلومات في الدول، والتي يمكن استخدامها لقياس أداء الرعاية الصحية الأولية.

**النتائج:**

كانت البيانات المتعلقة بنسبة %54 (32/59) من المؤشرات الكمية بشكل جزئي أو كلي للدول، وتراوحت من %41 (24/59) في باكستان إلى %64 (38/59) في نيبال. ويمكن الحصول على معلومات عن %41 (66/163) من المؤشرات الفرعية النوعية من خلال المراجعات المكتبية للوثائق الخاصة بكل دولة. وكانت المعلومات المتعلقة بمؤشرات المدخلات متوفرة بشكل أكثر جاهزية من المعلومات المتعلقة بمؤشرات العمليات والمخرجات. وبالنسبة للحصول على معلومات حول المؤشرات غير المبلغ عنها، كانت الجدوى من ذلك متوسطة إلى عالية من خلال تكييف أدوات جمع البيانات.

**الاستنتاج:**

إن إطار العمل الخاص بقياس الرعاية الصحية الأولية، يقدم منصة لتقييم وتتبع أداء الرعاية الصحية الأولية بشكل سهل. ينبغي على الدول تحسين اكتمال البيانات القائمة، وجودتها، واستخدامها من أجل تعزيز الرعاية الصحية الأولية.

## 摘要

**目的:**

旨在评估世界卫生组织和联合国儿童基金会初级卫生保健衡量框架指标相关信息在巴基斯坦、孟加拉国、尼泊尔、斯里兰卡和印度的可用性，并概述在这些国家使用该框架的机会及相关挑战。

**方法:**

我们仔细研究了全球和国家数据资源库以了解该框架的定量指标，并于 2023 年 2 月至 4 月针对国家文件进行了案头审查以了解定性指标。我们评估了数据来源和横断面调查工具，以推测当前在这些国家未报告的框架指标相关信息的可能来源。我们还确定了这些国家收集信息所依赖且可用于衡量初级卫生保健绩效的框架外具体指标。

**结果:**

54% (32/59) 的定量指标相关数据部分或完全可供这些国家使用，使用率从巴基斯坦的 41% (24/59) 到尼泊尔的 64% (38/59) 不等。可通过对国别文件进行案头审查获得 41% (66/163) 的定性子指标相关信息。投入指标相关信息比过程和产出指标相关信息更容易获得。通过调整数据收集工具，获取未报告指标相关信息的可行性介于一般和较高之间。

**结论:**

初级卫生保健衡量框架为有效评估和跟踪初级卫生保健绩效提供了平台。各国应提高现有数据的完整性、质量和利用率，以加强初级卫生保健。

## Резюме

**Цель:**

Оценить наличие информации о показателях системы измерения эффективности первичной медико-санитарной помощи Всемирной организации здравоохранения и Детского фонда Организации Объединенных Наций в Бангладеш, Индии, Непале, Пакистане и Шри-Ланке и обозначить возможности и проблемы использования этой системы в этих странах.

**Методы:**

С февраля по апрель 2023 года был проведен обзор глобальных и национальных хранилищ данных по количественным показателям структуры и аналитический обзор документов по странам по качественным показателям. Авторы проанализировали источники данных и инструменты проведения перекрестных исследований и сделали предположение о возможных источниках информации для рамочных показателей, которые в настоящее время не включаются в отчеты в указанных странах. Кроме того, были определены конкретные показатели, не входящие в рамочную схему, по которым в этих странах собирается информация и которые могут использоваться для оценки эффективности первичного медико-санитарного обслуживания.

**Результаты:**

Данные по 54% (32/59) количественных показателей были частично или полностью доступны по странам: от 41% (24/59) в Пакистане до 64% (38/59) в Непале. Информация по 41% (66/163) качественных подпоказателей была получена в результате анализа документов по конкретным странам. Информация о показателях затрат является более доступной, чем информация о показателях процессов и результатов. Возможность получения информации по показателям, не включенным в отчетность, оценивалась как умеренная или высокая в случае адаптации инструментов сбора данных.

**Вывод:**

Система статистического измерения показателей первичной медико-санитарной помощи обеспечивает платформу для удобной оценки и отслеживания эффективности первичного медико-санитарного обслуживания. В целях усиления первичной медико-санитарной помощи странам необходимо улучшить полноту, качество и использование доступных данных.

## Introduction

Health is central to the 2030 agenda of the United Nations (UN) sustainable development goals (SDG), and primary health care is a strategy to achieve most of the SDG health targets.^1^ The success of primary health care depends on consistent evaluation and performance review of service delivery, which provides strategic information for decision-making and builds accountability.^2^ Therefore, performance monitoring is essential to identify gaps, determine priorities, set targets, track progress, and guide actions and investments for overall improvement in population health, service coverage and financial risk protection against out-of-pocket expenditure.

Several frameworks have been developed to track the performance of primary health care in different regions of the world. The primary care assessment tool, developed between 1990 and 1999, provided a framework to evaluate service delivery processes.^3^ At the same time, the project Primary Health Care Activity Monitor for Europe assesses primary health-care structures such as governance, financing and workforce, along with processes and outcomes.^4^ However, these tools and other frameworks, such as the World Health Organization (WHO) European primary health-care impact, performance and capacity tool; the quality and outcomes framework (United Kingdom of Great Britain and Northern Ireland); and the results-based logic model for primary health care (Canada), have been designed for high-income countries and require modifications for use in low- and middle-income countries.^5^^,^^6^ In addition, while most of these frameworks capture the core primary health-care attributes of continuity, coordination, comprehensiveness, patient-centredness and quality of care, community engagement and multisectoral action are given limited attention.^7^

To bridge the gaps, the Primary Health Care Performance Initiative introduced a framework to assess primary health care in the low- and middle-income countries. This framework is built on a model specifying key inputs, processes, outputs and outcomes, and provides a scoring system for measuring primary health-care performance.^8^ Although the framework has adequate measures for components such as primary health-care spending, access, quality, service coverage and health outcomes, a few subdomains required improved validity of the indicators.^9^^–^^11^ A review of the literature on measurement of primary health-care performance in low- and middle-income countries highlighted the limited scope of existing primary health-care measurement frameworks. The review recommended the validation of existing indicators and the development of concise measures for the neglected dimensions of primary health care.^12^

In this regard, WHO and the United Nations Children’s Fund (UNICEF) developed an operational framework for measurement of primary health care through a technical review of existing frameworks and indicators, followed by multiple stages of stakeholder consultations.^13^ The framework has three components: integrated service delivery and essential public health functions; multisectoral policy and action; and empowered people and communities. The framework provides 87 indicators (59 quantitative and 28 qualitative), which are further classified as tier 1 (39 indicators) or tier 2 (48 indicators) based on the feasibility of their measurement. Tier 1 indicators are feasible to collect, monitor and track in most contexts, while tier 2 indicators are desirable to collect but acquiring the needed information may not be practicable in all contexts. Most of the indicators are composite indicators, information on which should be disaggregated by the sociodemographic drivers of equity. The novelty of the framework lies in the large set of lever-specific indicators, which can be tailored to national and subnational country contexts. The framework also provides guidance for measurement of particular areas, such as policy and governance, community engagement, organization, management of health services, and purchasing and payment systems.^14^

We aimed to assess first-hand experience of using the framework in five countries – Bangladesh, India, Nepal, Pakistan and Sri Lanka – to show the opportunities and challenges in evaluating primary health care. First, we assessed the availability of data on the indicators for evaluating primary health care in the countries. Second, we identified the data sources for the indicators and the level of disaggregation in these sources to track the equity drivers. Third, we identified opportunities to expand the scope of the existing data sources to provide information on the indicators for primary health-care monitoring that were not currently available. Finally, we propose a set of primary health-care monitoring indicators that can be added to the WHO-UNICEF primary health-care measurement framework based on information available in existing data sources in these countries.

## Methods

We used an exhaustive secondary data review complemented by stakeholder consultation. Based on the WHO-UNICEF primary health-care measurement framework, we assessed the availability of indicators for primary health-care monitoring in Bangladesh, India, Nepal, Pakistan and Sri Lanka. We searched for framework indicators in global data repositories during March–April 2023. These repositories included the WHO Global Health Observatory, WHO global health expenditure database, WHO health inequality monitor, World Bank indicator database, UN SDG indicator database, WHO national health workforce accounts, WHO state party assessment reports and WHO–UNICEF reports.^15^^–^^23^

We also searched national data repositories including national health accounts reports, health facility surveys, demographic and health surveys, noncommunicable disease monitoring surveys, national sample survey (India), national income and expenditure survey (Bangladesh) and multiple indicator cluster surveys (Bangladesh, Nepal and Pakistan), as well as the annual reports generated from data captured through the health management information systems.

We shared the list of indicators with relevant officials from the health ministry and development partners in India, Pakistan and Sri Lanka to identify the framework indicators on which information was being collected through some systems but which were not available in the public domain. We had access to tools used in routine information systems in Bangladesh and Nepal. We also conducted a desk review of country-specific documents. Finally, we organized a stakeholder consultation with 33 national and international experts from academia, government ministries, nongovernmental organizations and development partners, to ensure that we identified and included all relevant data sources to assess the availability of information on quantitative indicators as well as potential sources of information on the availability of qualitative indicators.

We analysed the percentage of quantitative indicators for which information was available by tier classification (feasibility of measurement) and the three components in the framework for each country. We also assessed the frequency of reporting of the data sources (cross-sectional surveys and routine management information systems), the proportion of reported indicators available, and the level of disaggregation of indicators compared with the level suggested by the primary health-care measurement framework.

In addition, we evaluated the country-specific cross-sectional survey tools, such as the service provision assessment tool, service availability and readiness assessment tool, demographic and health survey tools and noncommunicable disease monitoring survey tools, to identify potential framework indicators and subindicators that were unreported in the data repositories and reports. We further classified these indicators and subindicators into three categories according to the feasibility of data collection (easy, moderate and difficult). Data collection was considered easy if the question relevant to the indicator could be identified in the survey tools, but the information on the value of the indicator was not reported in the associated report. Moderately difficult data collection meant that the tool in its present form did not capture information on subindicators and would require modifications. Data collection was considered difficult if special surveys were required to collect information on the indicator. Indicators for which the feasibility of acquiring information on their subindicators varied were categorized according to the level of feasibility for at least half of the subindicators.

Finally, while reviewing the survey questionnaires, we also identified a set of indicators that are not currently included in the primary health-care measurement framework, but on which information is being collected and reported in the countries and which could be used to measure primary health-care performance in the region. 

### Ethical approval 

The Institute Ethics Committee at the Post Graduate Institute of Medical Education and Research, Chandigarh, India approved the study (PGI/IEC/2023/EIC000289).

## Results

### Availability of indicators

**Fig. 1:**
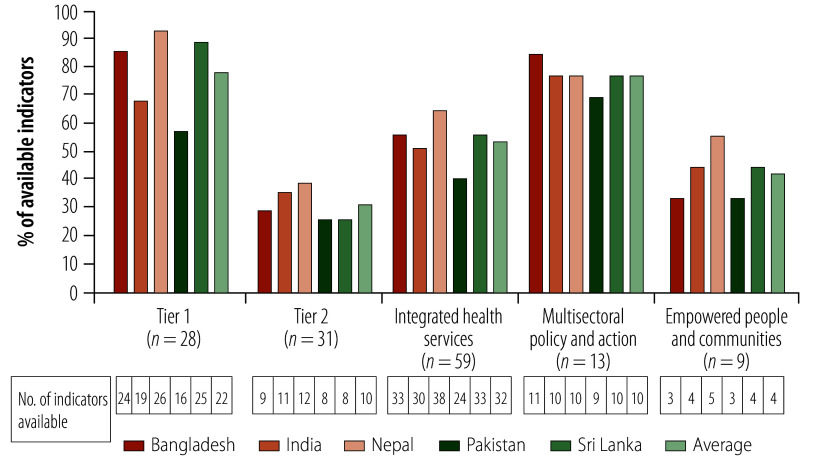
Proportion of available quantitative indicators of the WHO–UNICEF primary health care measurement framework, by country

UNICEF: United Nations Children’s Fund; WHO: World Health Organization.

On average, 54% (32/59) of the quantitative indicators were completely or partially available for the five countries, ranging from 41% (24/59) in Pakistan to 64% (38/59) in Nepal. Complete or partial information was available from the existing data sources for 79% (22/28) of tier 1 indicators, ranging from 57% (16/28) to 93% (26/28). For tier 2 indicators, information was available for 31% (10/31; inconsistency is due to rounding), ranging from 26% (8/31) to 39% (12/31; Fig. 1). Overall, information was available on 54% (32/59) of indicators related to integrated health services, ranging from 41% (24/59) to 64% (38/59), and 77% (10/13) of indicators related to multisectoral plan and action, ranging from 69% (9/13) to 85% (11/13). For empowered people and communities, information was available on 42% (4/9; inconsistency is due to rounding) of indicators, ranging from 33% (3/9) to 56% (5/9).

**Fig. 2:**
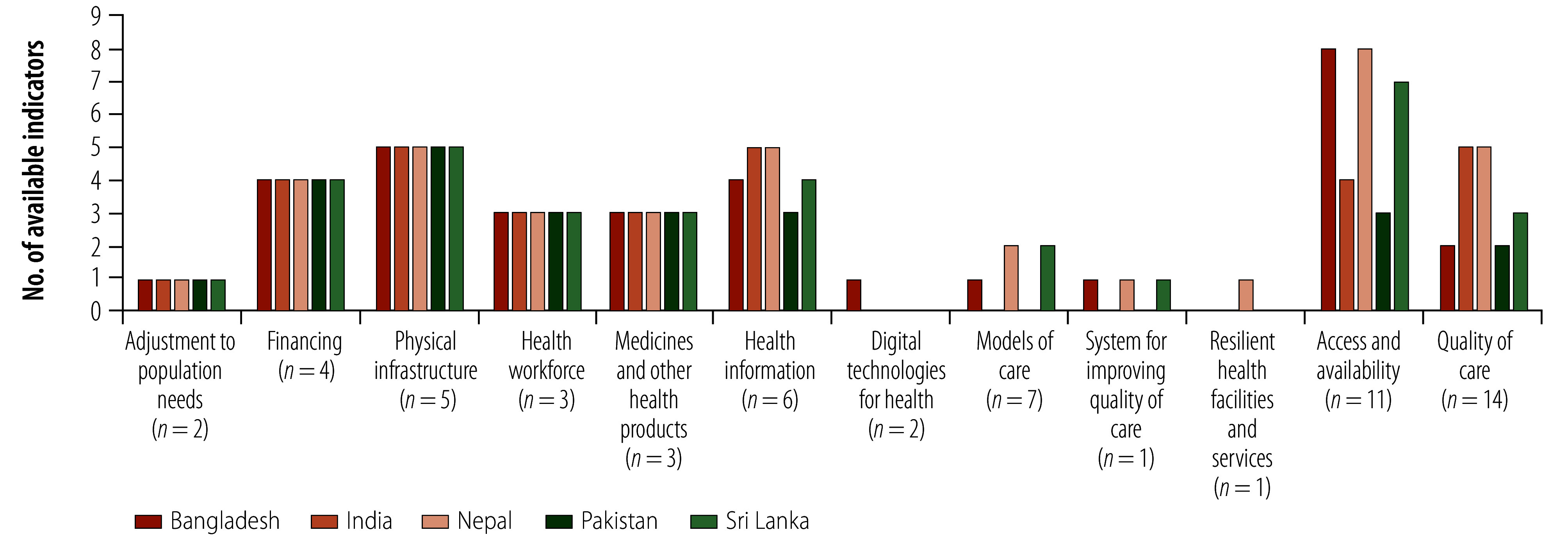
Available quantitative indicators of the WHO–UNICEF primary health care measurement framework, by domain

UNICEF: United Nations Children’s Fund; WHO: World Health Organization.

Note: *n* is the total number of indicators in each domain.

Bangladesh, Nepal and Sri Lanka reported information on more than 80% of tier 1 indicators, while the feasibility of obtaining information on tier 2 indicators using available data sources was highest for Nepal (39%; 12/31). Nepal had the highest percentage of quantitative indicators for which information was available: 64% (38/59) for integrated health services and 56% (5/9) for empowered people and communities. Bangladesh reported the highest percentage of indicators of multisectoral policy and action for which information was available (85%; 11/13; Fig. 1). Information on most of the input indicators was available for financing, physical infrastructure, health workforce, medicines and other health products (Fig. 2 and online repository).^24^ Limited information was available for models of care and output indicators, especially indicators measuring quality of care (Fig. 2; online repository).^24^

The framework divides the 28 qualitative indicators into 163 subindicators, of which information on 41% (66/163) could be acquired through desk reviews of country-specific documents, while 42% (69/163) required key informant interviews and 17% (28/163) needed both desk reviews and key informant interviews (online repository).^24^ Information on 61% (33/54) of the subindicators in the governance domain could be obtained from desk reviews of policy documents. Information on only 19% (8/42) of the subindicators for models of care could be acquired through the review of existing country-specific documents (online repository).^24^

### Data sources for quantitative indicators

Indicators on inputs and processes were mostly available from health facility surveys or facility censuses conducted by the countries. Data on outputs (utilization of services) were captured through the routine health information system in the countries. Global data repositories collated data to compute 11 quantitative indicators, while national surveys and routine information systems provided information for 29, 28, 33, 18 and 28 indicators in Bangladesh, India, Nepal, Pakistan and Sri Lanka, respectively.

**Fig. 3:**
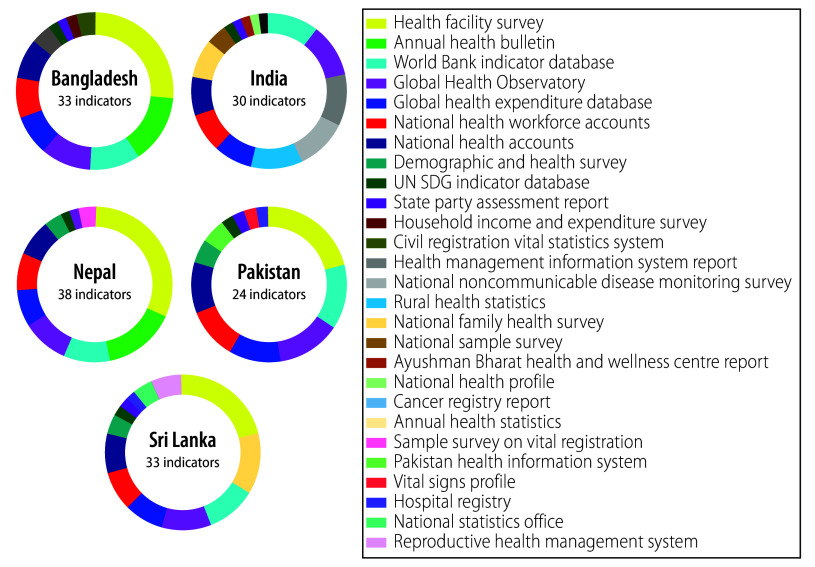
Proportion of available quantitative indicators of the WHO–UNICEF primary health care measurement framework, by data source and country

UNICEF: United Nations Children’s Fund; UN SDG: United Nations sustainable development goals; WHO: World Health Organization.

Note: the number of indicators is the total number of available indicators.

Health facility surveys were the source of information on the greatest number of indicators in all countries, except for India, followed by routine health information report, namely: annual health report in Nepal; annual health statistics in Sri Lanka; health management information system report in India; health information system in Pakistan; and annual health bulletin in Bangladesh (Fig. 3). Other surveys with large samples, such as demographic and health surveys and the national sample survey (India) provided information on only 2–5 quantitative indicators.

The methods or sources adopted by the countries to track indicators were similar in some cases and varied in others. For example, all the countries used national health accounts to track the finance-related indicators (Box 1; available at https://www.who.int/publications/journals/bulletin). Nepal and Sri Lanka recorded information on hospital discharges per 1000 population through the health information system, but Bangladesh and India captured this information through population-based surveys. While the information on indicators related to physical infrastructure and availability of human resources are collected annually in India through collation of information from government health facilities, other countries acquire these data through facility-based surveys. Box 1 provides the complete list of country data sources used for measuring various indicators.

Box 1 **Sources of information for quantitative indicators of the WHO–UNICEF primary health care measurement framework, Bangladesh, India, Nepal, Pakistan, Sri Lanka**

***Routine health information system^a^***

- Health facility density (Bangladesh, India, Nepal, Pakistan, Sri Lanka)
- Availability of basic water, sanitation and hygiene amenities (India)
- Facilities with power and communications (India)
- Outpatient visits (Bangladesh, India, Nepal, Sri Lanka)
- Emergency unit visits (Bangladesh, Nepal)
- Hospital discharges (Nepal, Sri Lanka)
- Leading diagnoses (Bangladesh, Nepal, Sri Lanka)
- Rate of admission with ambulatory care sensitive conditions (Bangladesh, Nepal, Sri Lanka)
- Provider caseload (India)
- Bed occupancy (Bangladesh, India, Nepal, Pakistan, Sri Lanka)
- Timely access (India, Pakistan, Sri Lanka)
- Percentage of completeness of reporting by facilities (India, Nepal)
- Telemedicine access (Bangladesh)

***Facility-based survey^b^; national noncommunicable disease monitoring survey^c^***

- Availability of basic water, sanitation and hygiene amenities (Bangladesh, Nepal, Sri Lanka)
- Facilities with power and communications (Bangladesh, Nepal, Sri Lanka)
- Availability of emergency transport (Bangladesh, India, Nepal, Sri Lanka)
- Availability of essential medicines (Bangladesh, India, Nepal, Sri Lanka)
- Availability of essential in vitro diagnostics (Bangladesh, India, Nepal, Sri Lanka)
- Availability of priority medical equipment and other devices (Bangladesh, India, Nepal, Sri Lanka)
- Existence of supportive supervision system (Bangladesh, Nepal)
- Facilities with systems to improve quality (Bangladesh, Nepal)
- Resilient health facilities and services (Nepal)
- Facilities offering services according to national defined service package (Bangladesh, Nepal, Sri Lanka)
- Percentage of facilities meeting minimum standards to deliver tracer services (Bangladesh, Nepal, Sri Lanka)
- Facilities compliant with infection prevention and control measures (Bangladesh, Nepal, Sri Lanka)
- Diagnostic accuracy (India)
- Patient-reported experiences (Nepal)
- Adherence to clinical standards for tracer services (Nepal)
- Proactive population outreach (Nepal)

***Population-based surveys (demographic and health survey and household income and expenditure survey^d^; national sample survey^e^)***

- Perceived barriers to access – geographical, financial, sociocultural (Bangladesh, India, Nepal, Pakistan, Sri Lanka)
- Hospital discharges (Bangladesh, India)
- Leading diagnoses (India)
- Patient-reported experiences (India)
- Completeness of birth registration (Bangladesh, India, Nepal, Pakistan, Sri Lanka)
- Completeness of death registration (India)

***National health accounts^f^***

- Current health expenditure on health (total and primary health care-specific) as percentage of GDP (Bangladesh, India, Nepal, Pakistan, Sri Lanka)
- Per capita total health expenditure, and primary health care-specific (Bangladesh, India, Nepal, Pakistan, Sri Lanka)
- Government primary health care spending as percentage of government health expenditure (Bangladesh, India, Nepal, Pakistan, Sri Lanka)
- Sources of expenditure on health, and primary health care-specific (Bangladesh, India, Nepal, Pakistan, Sri Lanka)

GDP: gross domestic product; UNICEF: United Nations Children’s Fund; WHO: World Health Organization.^a^ Bangladesh, India, Nepal, Pakistan and Sri Lanka.^b^ Bangladesh, Nepal and Sri Lanka.^c^ India.^d^ Bangladesh.^e^ India.^f^ Bangladesh, India, Nepal, Pakistan and Sri Lanka.

### Reporting and disaggregation

The average time between survey reports varied between countries. The average duration between rounds of demographic and health surveys ranged between 2 years for Bangladesh and 13.5 years for Sri Lanka (online repository).^24^^,^^25^ Similarly, health facility assessments were reported every 2 years for Bangladesh, 5 years for Nepal and 8 years for Pakistan.^26^^–^^29^ Additionally, the reporting of consecutive rounds was not consistent in the countries. For example, for the WHO STEPS (STEPwise approach to noncommunicable disease risk factor surveillance) surveys, while the first three rounds in Nepal had a gap of 1 year, the fourth and fifth rounds had a gap of 4 and 6 years, respectively.

**Fig. 4:**
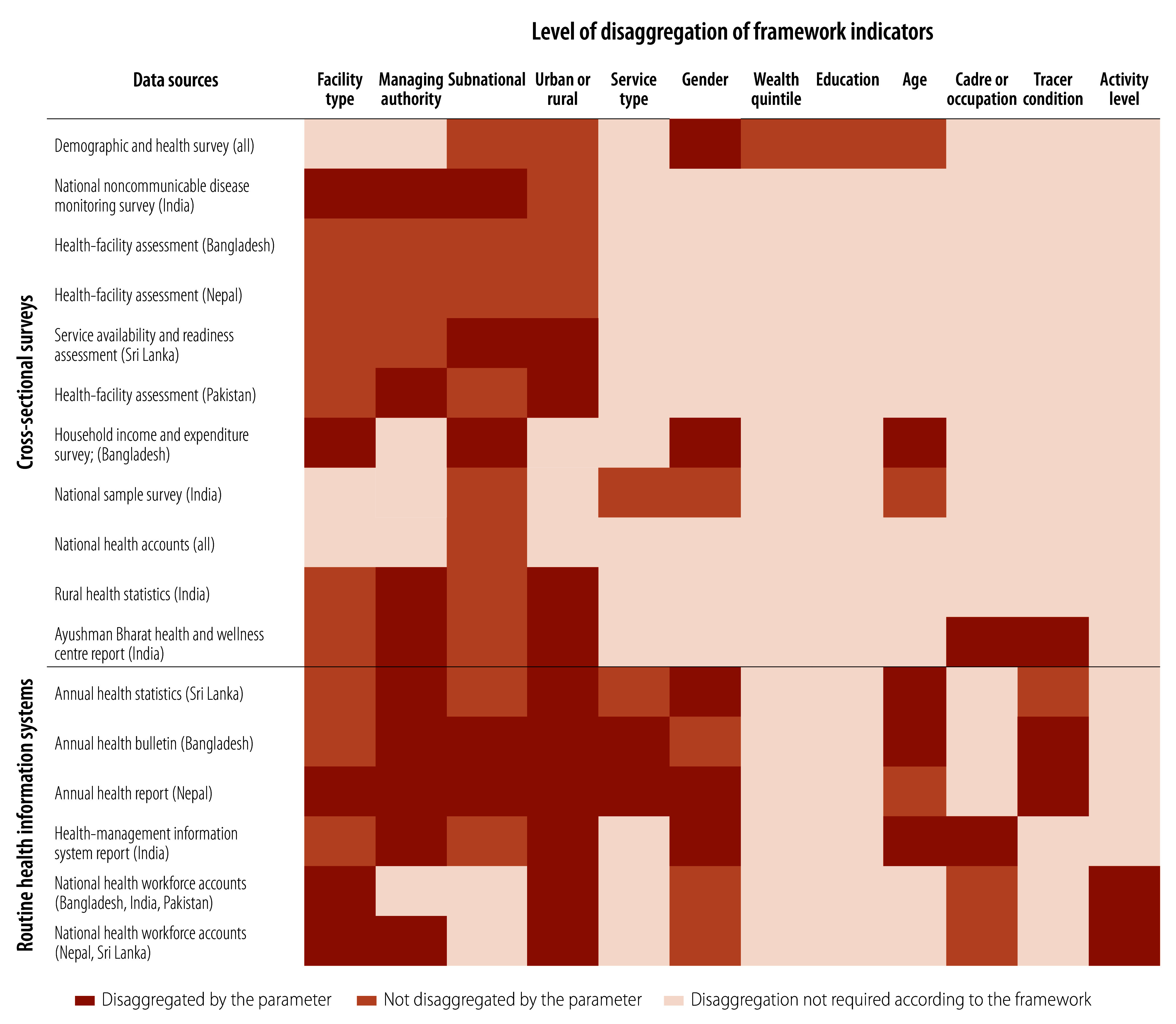
Level of disaggregation of indicators of the WHO–UNICEF primary health care measurement framework in the data sources

UNICEF: United Nations Children’s Fund; WHO: World Health Organization.

Note: details available in online repository.^24^

Health facility surveys in Bangladesh and Nepal disaggregate almost all the indicators according to facility type, managing authority, province or division and location (urban or rural), in line with the requirement of the framework (Fig. 4). The district health information system gathers information at the facility level which is later collated at district, provincial and national levels in the five countries. The routine information systems in all the countries do not collect data from private health providers, and do not report stratified results based on the location of the facilities.

### Potential data sources

Of 34 quantitative indicators on which information was unavailable, data on nine (26%) indicators can be acquired easily because of their inclusion in standard survey tools (Table 1). Overall, 59% (20/34) of the indicators were classified in the moderate category because data could be acquired by inclusion of related questions in the tools of existing surveys. The health systems in the countries are just starting to include some indicators, such as facilities using comprehensive patient records, facilities using electronic health records and subindicators (online repository).^24^

**Table 1:** Quantitative indicators of the WHO–UNICEF primary health care measurement framework, on which information is currently not available, potential data sources and ease of access of the data

Indicator	Potential data sources	Ease of data collection
Percentage of public research funding for primary research	National health accounts	Difficult
Health-worker density and distribution	National health workforce accounts	Moderate
Accreditation mechanisms for education and training institutions	National health workforce accounts, regulatory councils	Moderate
National system for continuing professional development	National health workforce accounts, regulatory councils	Moderate
Availability of medicines	Health facility surveys	Easy
Availability of essential in vitro diagnostics	Health facility surveys	Moderate
Availability of priority medical equipment and other medical devices	Health facility surveys	Moderate
Percentage of facilities using comprehensive patient records	Health facility surveys	Difficult
Functional national human resources information system and national health workforce accounts	Desk reviews of policy documents	Easy
Percentage of facilities using electronic health records	Health facility surveys	Difficult
Management capability and leadership: percentage of facilities with managers or teams that have decision-making responsibilities	Health facility surveys	Easy
Multidisciplinary team-based service delivery^a^	Health facility surveys, key informant interviews, routine health information systems	Difficult
Existence of facility budgets and expenditures meeting criteria	Health facility surveys	Moderate
Collaboration between facility-based and community-based service providers^a^	Policy reviews, health facility surveys, routine health information systems	Moderate
Proactive population outreach	Health facility surveys	Moderate
Services for self-care and health literacy in primary care	Health facility surveys	Moderate
Percentage of health facilities with systems to support improvements in quality^a^	Health facility surveys	Easy
Percentage of facilities meeting criteria for resilient health facilities and services^a^	Health facility surveys	Moderate
Geographical access to services	Geographic information system mapping	Easy
Access to emergency surgery	Population-based surveys	Easy
Percentage of facilities offering services according to a national service package	Health facility surveys	Moderate
Provider availability (absence rate)	Health facility surveys	Moderate
Percentage of facilities meeting minimum standards to deliver tracer services	Health facility surveys	Moderate
Percentage of facilities compliant with infection prevention and control measures	Health facility surveys	Moderate
Patient-reported experiences^a^	Population-based surveys, health facility surveys	Moderate
People’s perception of health system and services	Population-based surveys, health facility surveys	Easy
Diagnostic accuracy (provider knowledge)	Health facility survey	Easy
Adherence to clinical standards for tracer conditions	Health-facility surveys	Easy
30-day hospital case fatality rate (acute myocardial infarction or stroke)	Routine health information systems	Moderate
Avoidable complications (lower limb amputation in diabetes)	Routine health information systems	Moderate
Hospital readmission rate for tracer conditions	Routine health information systems	Moderate
Prescribing practices for antibiotics	Prescription audit, health facility surveys	Moderate
Proportion of people 65 years and older prescribed antipsychotics	Prescription audit, health facility surveys	Moderate
Waiting time for elective surgery	Population-based surveys, health facility surveys	Difficult

^a^ Indicator has subindicators that had different levels of feasibility of data collection (online repository).^24^

The framework suggests that data be collected on a few subindicators of the quantitative indicator called multidisciplinary team-based service delivery. However, some components, such as team identity, clearly defined roles and responsibilities that are uniformly understood by all team members, shared goals of providing quality care, and mutual accountability structures, require a qualitative investigation in the region (online repository).^24^ Additionally, the framework recommends both qualitative and quantitative assessment for a few indicators, including access to telemedicine, existence of an empanelment system and facilities with systems to support quality improvement. We found that these indicators were being evaluated quantitatively through existing data repositories in at least one of the five countries.

### Extending the framework

Information on some indicators related to multisectoral coordination is readily available in the five countries, such as: number of people stopped and checked by traffic police for drink–driving in the past 12 months in Nepal; number of training programmes conducted for ministry of health and non-ministry of health personnel in Bangladesh; population being taught in school and college about the ill-effects of tobacco and alcohol, and the benefits of physical activity and diet in India; and use of safety helmets by motorcycle drivers and passengers in Nepal and Pakistan (Box 2; available at https://www.who.int/publications/journals/bulletin). Additionally, several indicators related to community links and engagement, such as number of committee meetings held with at least 60% of members, awareness of community outreach activities, and population with recent contacts with health workers, were collected by the countries on a regular basis (Box 2; online repository).^24^

Box 2 **Additional primary health care monitoring indicators being tracked, WHO–UNICEF primary health care measurement framework, Bangladesh, India, Nepal, Pakistan, Sri Lanka**

**Governance**

- Percentage of adolescents reported being taught in school and/or college about ill-effects of tobacco and alcohol (India)
- Percentage of adults (motorcycle drivers and passengers) using safety helmets while being on a motorcycle or scooter in past 30 days (Nepal, Pakistan)
- Percentage of adults exposed to anti-tobacco messages through various forms of the media (Nepal, Pakistan)
- Percentage of adults driving a vehicle in past 12 months reported to be stopped or checked by traffic police for drink–driving (Nepal)
- Number of *upazilas* (administrative division in Bangladesh) covered for staff training on animal control (Bangladesh)

**Information system and digital technologies**

- Percentage of facilities that have a designated focal person for the integrated health management information system (Nepal)
- Number of participants in local training on management information systems (Bangladesh)
- Number of people calling to *Shastho Batayon* (teleconsultation service) for health problems (Bangladesh)
- Number of teleconsultations (India)
- Number of *Ayushman Bharat* health account numbers (unique health identifier) issued (India)

**Accessibility, affordability acceptability (perceived barriers to access)**

- Percentage of adults 15–69 years with existing oral health issues reporting reasons for not seeking care for these issues (Nepal)
- Percentage of people 15–69 years advised to take medicines but have not taken these medicines in the past 2 weeks, and reasons for not taking medication for high blood pressure or diabetes (Bangladesh, Nepal, Sri Lanka)
- Percentage of men aged 15–49 years whose youngest living child was 0–35 months reporting the reasons why the child’s mother did not receive antenatal care when pregnant (India)
- Among households not generally using a government health facility when their members are sick, percentage giving reasons for not using government health services (India)
- Percentage of the population with a health facility within 5 km of their home (Bangladesh, Sri Lanka)

**Organization and facility management**

- Percentage of facilities with management committees where committee members meet at least once every 6 months (Nepal)
- Percentage of facilities with supervisory visit 4 months before the health facility survey (Nepal)
- Percentage of facilities having completed financial audits (Nepal)

**Community linkages and engagement**

- Number of individuals counselled on nutrition, substance abuse, reproductive tract infections and/or sexually transmitted infections (India)
- Number of mothers attending mother’s health group meetings (Nepal)
- Percentage of mothers with children 0–4 years who read books given by the public health midwives or family health officer before or after the birth of their last child (Sri Lanka)
- Percentage of newborn admissions in special newborn care units referred by an accredited social health activist (village-level community health workers) (India)
- Number of monthly *Jan Aarogya Samiti* (facility management committee) meetings held with at least 60% of members (India)

**Systems for improving quality**

- Percentage of facilities with system for determining client opinion (Nepal, Sri Lanka)
- Number of certified adolescent-friendly sites (Nepal)
- Average minimum service standards scores for hospitals (Nepal)
- Number of cases of adverse events following immunization (India, Sri Lanka)
- Percentage of facilities with system for determining client opinions and procedure for reviewing client opinions (Bangladesh)
- Completion rate of preliminary investigations of complaints within 1 month of reporting (Sri Lanka)

**Service availability and readiness**

- Percentage of facilities with capacity to process equipment for reuse – adherence to standards for quality sterilization (Nepal)
- Number of facilities functioning day and night 7 days a week (India)
- Percentage of facilities using different methods, such as incinerator, open burning, dump without burning, offsite removal, for managing waste (Bangladesh)
- Percentage of facilities offering any modern family planning method that have items for infection control (Bangladesh)

**Resilient health facilities and services**

- Percentage of facilities with an outbreak management plan (Nepal)
- Percentage of facilities with a mass casualty management plan (Nepal)

**Core primary care functions (patient-reported experiences)**

- Percentage of caretakers who considered specific service issues to be a major problem for them on the day of their visit (Nepal)
- Percentage of family planning clients who considered specific service issues to be a major problem for them on the day of their visit (Nepal)
- Percentage of postpartum women reporting waiting times to see a provider (Nepal)
- Percentage of postpartum women who reported satisfaction with waiting time, information received from providers, staff behaviour, cleanliness, level of privacy, and care received (Nepal)
- Percentage of complaints by category in complaints and suggestion system (Bangladesh)

**Core primary care functions (people-centredness)**

- Percentage of postpartum women reporting respectful maternity care (Nepal)
- Percentage of current users of contraceptive methods (women aged 15–49 years) informed about possible side-effects, what to do if they experienced side-effects and other methods they could use (Nepal)
- Among all female family planning clients with observed consultations, percentage whose consultation included the indicated components and the indicated discussions related to sexually transmitted infections and condoms (Nepal)
- Percentage of ever-married women aware of service providers (Sri Lanka)
- Percentage of facilities with private spaces available during consultations (Bangladesh)

**Utilization of services**

- Percentage of people 15–69 years ever told they had raised blood pressure or blood sugar and mentioning receiving advice on hypertension and diabetes (Nepal, Bangladesh, Sri Lanka)
- Percentage of adults 30–69 years diagnosed with known cardiovascular conditions in a government health facility (India)
- Percentage of adolescents receiving clinical and counselling services at adolescent-friendly health centres (India)
- Percentage of live births in the 3 or 5 years preceding the demographic and health survey by place of delivery – home or health facility (India, Pakistan, Sri Lanka, Nepal, Bangladesh)
- Percentage of children younger than 5 years with symptoms of acute respiratory infection 2 weeks before the survey for whom advice and treatment was sought from specific sources (Pakistan)

**Proactive population outreach**

- Number of clients receiving services provided by outreach clinics, such as primary treatment, antenatal and postnatal care, condoms and contraceptive pills (Nepal)
- Number of women receiving a postnatal care visit within 2 days of delivery (Nepal)
- Number of village health and nutrition days held (India)
- Number of community-based assessment checklists filled (India)
- Percentage of women aged 15–49 years discussing family planning with a field worker in the 6 months before the survey (Bangladesh)

**Efficiency**

- Number of drug quality failures (events) reported during a quarter (Sri Lanka)
- Cost of discarded drugs (Sri Lanka)
- Percentage use of annual financial allocation (Sri Lanka)

Only a few indicators for each domain of the framework that are being tracked in the region are presented. See online repository for complete lists of indicators and country-specific data sources.^24^

## Discussion

Our paper presents information on the current availability of indicators within the primary health-care measurement framework in five countries and suggests the extension of existing surveys to obtain information, especially related to process and output indicators. Overall, complete or partial information was available for four fifths and one third of the of the tier 1 and tier 2 quantitative indicators, respectively, from the existing data sources. Most of the information was available for quantitative indicators related to multisectoral plan and action, and integrated health services. However, information on the indicators related to empowered people and communities need to be strengthened to compute the indicator. Data availability was skewed towards input indicators, in comparison to process, and output indicators in all the countries. Information was not available for about a quarter of the quantitative indicators, even though this information is included in standard survey tools and the data could be easily obtained. Thus, countries have data on such indicators, which, with adequate reporting, can be used for performance evaluation.

Countries used different approaches to track certain indicators, using either surveys or routine information systems. Both methods of data collection have advantages as well as limitations. While data collected through management information systems are timely and present a more sustainable option for performance monitoring of primary health care, they depend on the completeness of reporting by facilities and currently do not include information from private entities. Moreover, ensuring the quality of data from management information systems is important for estimating performance. On the other hand, evidence collected from cross-sectional surveys is reliant on self-reported information. While these surveys may not have problems of over-reporting or lack of representation of the private sector, their sample sizes generally do not permit district-level analysis. Stakeholders may prioritize data sources based on available resources and local capacities; however, robust mechanisms should be in place to ensure data quality. 

Governments should not only consider building systems for improvement in availability of standardized data for monitoring primary health-care performance, but also adapt the framework and use indicators on which information is readily available to ensure context-specific monitoring systems. The countries have adopted similar approaches to establish community participation through institutionalization of health committees and peer groups, and through community health workers.^30^^–^^32^ Therefore, indicators already being tracked in the countries may be useful to assess the level of community engagement in service planning and organization after consideration of their appropriateness. 

Furthermore, with the increasing burden of noncommunicable diseases,^33^ the success of primary health care will be contingent on the continuum of care provided. In this regard, the framework suggests assessment of the existence of comprehensive patient records and the availability of protocols for referrals, back-referrals, and emergency transfer, but does not include output indicators for assessment of the care continuum. However, in Bangladesh and India, authorities have started to track the number of upward and downward referrals through routine management information systems. In one of the surveys on primary health-care facilities in India, the population-level compliance rate with referrals made by the primary health-care teams was also assessed.^34^

The so-called leave no-one behind principle requires monitoring of indicators with detailed disaggregation across population subgroups, regions and levels of care. Most of the sample surveys presented disaggregated data on certain parameters, as suggested by the framework. However, the reports of management information systems contained limited disaggregation. The framework recommends disaggregation of 58% of quantitative indicators by facility type, which implies measurement of primary health care delivered at primary, secondary and tertiary level facilities. The measurement of indicators at secondary and tertiary level facilities should be analysed with caution in the studied countries as no formal gatekeeping mechanism exists at primary health-care facilities. Thus, the aggregated measurement of primary health care at all three levels of health care is likely to overestimate the actual availability of resources for primary health-care service delivery and primary health-care performance at the designated primary health-care facilities. This situation is likely to promote an inefficient model of primary health-care service delivery.^35^^,^^36^ Disaggregation of these data may be useful to monitor care-seeking patterns and to quantify the effectiveness of any gatekeeping mechanism in strengthening primary health care. Moreover, the private sector in the region provides an estimated 50–69% of the outpatient care. Therefore, creating a mechanism for collecting data from private providers is crucial to increase the representativeness of data.

To improve the availability and accessibility of information across countries, global repositories are consistently being updated. The expansion of information in the national health workforce accounts and global health expenditure database, and the launch of a health inequality online repository in April 2023 are a few examples of the efforts being made globally to improve transparency and tracking of primary health-care performance.^16^^,^^17^^,^^20^ In addition, it is important to strengthen civil registration and vital statistics systems to capture, among other information, reliable data on cause of death for effective primary health-care service planning and delivery.^37^ Additionally, strengthening coordination and interoperability within and between different levels of care will also be essential to measure continuity of care and care coordination.

Our study has some limitations. First, the five countries have vertical programmes for certain diseases, with isolated reporting lines, which may not be integrated into a regular management information system. We did not evaluate reports of such programmes to identify domain-specific indicators not mentioned in the framework but being tracked in the countries. Second, reports of health information systems for Pakistan could not be directly accessed for the analysis, which may have resulted in under-representation of information on indicators outside the framework. Third, because information on data sources for qualitative indicators across the countries varied in rigour, the availability of information on qualitative indicators could not be analysed by country and requires further exploration. Finally, our assessment focused specifically on the five countries; however, extending such analyses to other countries in the region would help determine the generalizability of the findings.

In conclusion, the WHO–UNICEF primary health-care measurement framework provides an opportunity to set up a unified monitoring system for tracking the performance of primary health care. With different health system structures in urban areas and a substantial amount of care delivered by private providers in the region, data on the indicators should be collected and analysed according to these parameters. The focus of the countries in future should not only be to establish mechanisms for collection of information on the indicators for measurement of primary health-care performance, but also to work on improving the completeness, quality and use of existing data for strengthening of primary health care.
